# DNA Copy Number Profiling in Extracellular Vesicles as Clinical Biomarkers of High‐Grade Serous Ovarian Carcinoma

**DOI:** 10.1002/jev2.70308

**Published:** 2026-05-15

**Authors:** Ryosuke Uekusa, Akira Yokoi, Mayu Ukai, Kosuke Yoshida, Kunanon Chattrairat, Takao Yasui, Yasuhide Inokuma, Masami Kitagawa, Eri Asano‐Inami, Masato Yoshihara, Satoshi Tamauchi, Nobuhisa Yoshikawa, Kaoru Niimi, Juntaro Matsuzaki, Takahiro Ochiya, Anil K. Sood, Yusuke Yamamoto, Hiroaki Kajiyama

**Affiliations:** ^1^ Department of Obstetrics and Gynecology Nagoya University Graduate School of Medicine Nagoya Japan; ^2^ Nagoya University Institute For Advanced Research Nagoya Japan; ^3^ Japan Science and Technology Agency (JST) Kawaguchi Japan; ^4^ Department of Gynecologic Oncology Hyogo Cancer Center Akashi Hyogo Japan; ^5^ Department of Life Science and Technology Institute of Science Tokyo Yokohama Japan; ^6^ Division of Applied Chemistry, Faculty of Engineering Hokkaido University Sapporo Hokkaido Japan; ^7^ Institute For Chemical Reaction Design and Discovery (WPI‐ICReDD) Hokkaido University Sapporo Hokkaido Japan; ^8^ Division of Pharmacotherapeutics Keio University Faculty of Pharmacy Tokyo Japan; ^9^ Department of Molecular and Cellular Medicine Institute of Medical Science, Tokyo Medical University Tokyo Japan; ^10^ Department of Gynecologic Oncology and Reproductive Medicine University of Texas MD Anderson Cancer Center Houston Texas USA; ^11^ Center For RNA Interference and Non‐Coding RNA University of Texas MD Anderson Cancer Center Houston Texas USA; ^12^ Laboratory of Integrative Oncology National Cancer Center Research Institute Tokyo Japan

**Keywords:** copy number variations, extracellular vesicles, exosomes, EV‐DNA, high‐grade serous ovarian carcinoma

## Abstract

Extracellular vesicles (EVs), including exosomes, circulate in body fluids and carry pathological genomic information. High‐grade serous ovarian carcinoma (HGSOC) is the most common subtype of ovarian cancer, characterized predominantly by copy number variations (CNVs). This study evaluated the clinical significance of EV‐DNA focusing on CNV profiles and exploring its potential as a companion biomarker for predicting therapeutic response and supporting diagnosis in HGSOC. Droplet digital polymerase chain reaction detected concordant CNVs between tumor DNA and EV‐DNA in ascites but not in serum. EV‐DNA showed higher concordance with the CNV status of tumor DNA than cell‐free DNA in ascites. The EV‐DNA amount in malignant ascites was significantly higher than that in ascites from benign ovarian tumors (*p* < 0.05), and elevated EV‐DNA was observed even in cytology‐negative ovarian cancer, suggesting an increase early in disease development. Moreover, CNV profiles showed marked differences between responders and nonresponders to poly(ADP‐ribose) polymerase (PARP) inhibitors. An equation based on five genes (*ARID1A, NOTCH3, CSMD3, ELP4*, and *BARD1*) showed strong predictive performance for olaparib response (area under the curve = 0.91). Collectively, these findings indicate that the CNV status of EV‐DNA may serve as a non‐invasive companion biomarker for patient stratification and therapeutic monitoring in HGSOC.

## 1 Introduction

Ovarian cancer is the third most common gynecologic malignancy and the second leading cause of gynecologic cancer mortality worldwide in 2022^1^. High‐grade serous ovarian carcinoma (HGSOC), the most common subtype, is often diagnosed at advanced stages; hence, HGSOC has a high mortality rate. The estimated 5‐year survival rate of this malignancy is ∼50%. Despite an ∼80% response rate to standard treatment, including optimal debulking surgery and platinum‐based chemotherapy, most patients develop disease recurrence and progression within 2 years. Patients with ovarian cancer can experience multiple recurrences, which gradually develop into platinum resistance (Kurnit et al. 2021; Pignata et al. 2017). Poly(ADP‐ribose) polymerase (PARP) inhibitors have brought significant advancement in treating advanced ovarian cancer in recent years and are now key drugs for maintenance therapy (Lheureux et al. 2019). The gold standard biomarker for PARP inhibitors is homologous recombination (HR) repair defect status, including BRCA mutations, whereas that for its clinical biomarker is platinum‐sensitive recurrent status (Jiang et al. 2019; Mirza et al. 2020). However, these biomarkers often do not reflect clinical situations; thus, the need for new biomarkers is increasing. According to the previous report, genomic data of various cancer types from The Cancer Genome Atlas (TCGA) were analyzed, and the dominant genomic alterations of HGSOC were *TP53* mutation and copy number variations (CNVs) (Ciriello et al. 2013). Furthermore, Ovarian Cancer Moon Shot (OCMS) project has revealed dominant CNV‐related genes in HGSOC (Lee et al. 2020). Despite the genomic significance of CNVs in ovarian cancer, noninvasive CNV assessments as biomarkers have not been investigated, and most genomic biomarkers in body fluids focus on just the amount of DNA or specific mutations.

In general, extracellular vesicles (EVs) form a bilayer lipid membrane and are released from all living cells. EVs contain diverse bioactive molecules, including DNA, crucial in cell‐to‐cell communication (Colombo et al. 2014, Yokoi and Ochiya 2021). Small EVs (sEVs) have a diameter of <200 nm, whereas medium/large EVs (m/lEVs) have a diameter of ≥200 nm (Gould and Raposo 2013, Welsh et al. 2024). In oncology, cancer cells release EVs that affect nearby cells and distant sites, creating a cancer microenvironment that subsequently promotes cancer invasion and metastasis (Azmi et al. 2013; Hoshino et al. 2015; Yang and Robbins 2011). Because EVs are stable and widely exist in body fluids, including blood, urine, and ascites, EVs have the potential as a biomarker for diagnosis and prognosis (Lane et al. 2018; Urabe et al. 2020). The presence of DNA in EVs was first reported in 2014, reflecting the genetic information of the tumor tissue (Thakur et al. 2014). As for ovarian cancer, EV‐based biomarker studies have mostly focused on RNA and proteins (Chang et al. 2019; De Giorgis et al. 2024, Li et al. 2023), whereas few have focused on DNA. Our group previously reported the mechanism that cancer cells positively secrete DNA in EVs via micronuclei, which is a sign of genomic instability (Yokoi et al. 2019). However, CNV profiling in EVs was not fully assessed, and the clinical utility of EV‐DNA remains largely unexplored.

This study demonstrated that tumor CNV status can be detected through EV‐DNA analysis, and CNV analysis of EV‐DNA could serve as a diagnostic and predictive biomarker. Tumor CNV status could be detected in EV‐DNA in ascites using digital polymerase chain reaction (PCR). The presence of EV‐DNA in ascites suggested the existence of malignant diseases. Moreover, CNV analysis could predict response to PARP inhibitor treatment. These results provide new insights into liquid biopsy for HGSOC.

## 2 Materials and Methods

### 2.1 Cell Lines

Human ovarian cancer cell lines were obtained from the American Type Culture Collection (ATCC), European Collection of Cell Cultures (ECACC), and Japanese Collection of Research Bioresources (JCRB). CAOV3, OVCAR3, and OV90 cells were purchased from the ATCC. COV362 and PEO‐1 cells were from the ECACC. Kuramochi cells were from the JCRB. All cell lines were cultured in an optimal medium according to the suppliers’ recommendations. All cells were grown in humidified incubators at 37°C with 5% CO_2_.

### 2.2 Patient Selection

To predict the PARP inhibitor response, patients with HGSOC treated with olaparib at Nagoya University Hospital from 2018 to 2023 were collected (*n* = 70). Thirty‐three patients who received the drug continuously for >6 months and nine patients who discontinued the drug within 6 months due to disease recurrence or progression were selected. Patient characteristics are shown in Table 1. DNA was extracted from formalin‐fixed, paraffin‐embedded (FFPE) tissue of ovarian cancer at the time of surgery. This study was approved by the Ethics Committee of Nagoya University (approval no. 2013–0078), and informed consent was obtained from all participants.

**TABLE 1:** Patient characteristics for CNV analysis from FFPE tissue treated with olaparib for maintenance therapy.

	Pt. number	Age	Duration (months)	Sample collection	FIGO stage (2014)	Somatic *BRCA* mutation	HRD
Responders	1	59	76	Exploratory laparotomy	IIIC	Positive	—
	2	56	64	SDS (after chemotherapy)	IIIC	Negative	—
	3	72	62	SDS (after chemotherapy)	IA	Negative	—
	4	49	55	IDS	IVA	Negative	—
	5	52	54	IDS	IIIC	Positive	—
	6	45	46	PDS	IIIA	Positive	—
	7	72	44	IDS	IIIB	Positive	—
	8	55	43	IDS	IVB	Positive	—
	9	63	33	PDS	IVB	Positive	—
	10	59	31	Exploratory laparotomy	IIIB	Positive	Positive
	11	63	28	PDS	IIA	Negative	—
	12	59	27	IDS	IIIC	Negative	Positive
	13	42	27	SDS (after chemotherapy)	IIIB	Positive	—
	14	61	27	Exploratory laparotomy	IIIC	Negative	—
	15	68	25	IDS	IVA	Negative	—
	16	33	24	Exploratory laparotomy	IIIC	Positive	—
	17	41	24	Exploratory laparotomy	IIIC	—	Positive
	18	61	24	biopsy	IVB	Negative	Positive
	19	64	24	IDS	IIIC	Positive	Positive
	20	64	20	PDS	IIIC	Positive	Positive
	21	50	20	biopsy	IVB	—	—
	22	68	16	SDS (after chemotherapy)	IIIC	Negative	Positive
	23	56	14	PDS	IVB	—	Negative
	24	41	14	Exploratory laparotomy	IIIC	Positive	Negative
	25	71	11	PDS	IIIB	Negative	—
	26	56	9	PDS	IIIC	Negative	—
	27	38	8	IDS	IVB	Negative	Negative
	28	43	8	IDS	IVA	Negative	Negative
	29	59	8	IDS	IIIC	—	—
	30	63	7	IDS	IIIC	Negative	—
	31	66	7	Exploratory laparotomy	IIIC	Negative	Positive
	32	57	7	PDS	IIIC	Positive	—
	33	69	6	IDS	IIIC	Negative	Negative
Nonresponders	34	73	3	Exploratory laparotomy	IIIC	Negative	—
	35	63	3	IDS	IIIC	Negative	—
	36	53	3	IDS	IVB	Negative	—
	37	54	2	IDS	IIIC	—	Negative
	38	38	2	PDS	IIIC	Negative	—
	39	77	2	Exploratory laparotomy	IIIC	—	—
	40	60	1	Exploratory laparotomy	IIIC	Negative	—
	41	70	1	PDS	IIIC	Negative	—
	42	60	1	IDS	IIIC	Negative	—

Abbreviations: HRD, Homologous Recombination Deficiency; IDS, interval debulking surgery; PDS, primary debulking surgery; SDS, secondary debulking surgery.

For CNV evaluation of normal ovarian tissue, 15 patients who underwent surgery for benign ovarian tumors at our hospital were selected. Patient characteristics are shown in Table S1. DNA was extracted from FFPE tissue of contralateral normal ovarian tissue obtained at surgery for benign ovarian tumors.

For DNA analysis in ascites based on ascites cytology results, 26 patients with benign ovarian tumors, 32 patients with ascites cytology‐negative ovarian cancer, and 36 patients with ascites cytology‐positive ovarian cancer were selected from patients operated on in our hospital. The group with negative ascitic cytology included nine cases classified as suspicious for malignancy. Patients’ characteristics are shown in Table S2. EVs were extracted from 1 mL of undiluted ascites at the time of surgery.

HR status was examined using the MyChoice CDx HRD Companion Diagnostic Test (Myriad Genetics, Inc., Salt Lake City, UT, USA).

### 2.3 Isolation of EVs and Analysis

The EV isolation method used in this study followed the standard indications of the International Society for EVs (Welsh et al. 2024, Witwer et al. 2013).

Cells were washed with phosphate‐buffered saline (PBS), and the medium was replaced with advanced RPMI 1640 (Thermo Fisher Scientific, Waltham, MA, USA) for 48 h to collect the conditioned medium (CM). Advanced Dulbecco's modified Eagle's medium (Thermo Fisher Scientific) was used for COV362 cells. CM was centrifuged at 500 × *g* for 15 min at 4°C to remove cellular debris. CM was centrifuged at 10,000 × *g* for 40 min at 4°C. The pellets were washed with Dulbecco's PBS (D‐PBS) and centrifuged similarly at 10,000 × *g* for 40 min at 4°C. The pellet was resuspended in PBS and stored at 4°C as m/lEVs. The CM supernatant from the previous centrifugation was filtered through a 0.22 µm filter and centrifuged at 32,000 rpm using an SW32Ti rotor (Beckman Coulter, Inc., Brea, CA, USA) for 2 h at 4°C. The pellets were washed with PBS and centrifuged similarly at 32,000 rpm for 2 h at 4°C. The pellet was resuspended in PBS and stored at 4°C as sEVs.

To isolate EVs from serum and ascites obtained from patients with ovarian cancer, 1 mL of each patient's serum or ascites sample was used. Serum samples were processed according to standardized institutional procedures. Blood samples were allowed to clot and centrifuged on the same day of collection, and the resulting serum was collected and handled under controlled conditions according to a predefined protocol. Each sample was centrifuged at 500 × *g* for 15 min at room temperature to remove debris. The supernatant was centrifuged at 10,000 × *g* for 40 min at 4°C. The pellet was resuspended and centrifuged similarly at 10,000 × *g* for 40 min at 4°C. The pellet was resuspended in PBS and stored at 4°C as m/lEVs. The supernatant was filtered through a 0.22 µm filter to remove any remaining large vesicles. After filtration, the medium was centrifuged at 55,000 rpm using a TLA‐55 rotor (Beckman Coulter) for 120 min at 4°C in an ultracentrifuge. The pellet was resuspended to PBS and centrifuged similarly at 55,000 rpm for 120 min at 4°C. The final pellet was resuspended in PBS and stored at 4°C as sEVs.

The NanoSight system (Quantum Design Japan, Inc., Tokyo, Japan) was used to carry out a nanoparticle tracking analysis and calculate particle size using the Stokes‐Einstein equation to assess the size distribution of EVs. Nanoparticle tracking analysis was performed in duplicate measurements for each sample, and representative data are shown.

### 2.4 Transmission Electron Microscopy (TEM)

Samples of sEVs and m/lEVs derived from Kuramochi cells and ascites and serum were used for visualization. A mesh grid with carbon support film (Nissin‐EM, Tokyo, Japan) was hydrophilized with a hydrophilic treatment device (PIB‐10, vacuum device, Mito, Japan). The support membrane surface was attached to a 5 µL drop of the EV sample for 10 s. The sample was blocked with 1% bovine serum albumin for 1 h, washed five times with D‐PBS, fixed with 1% glutaraldehyde for 10 min, washed five times with ultrapure water, and stained with uranium for 10 s. The samples were dried thoroughly and visualized by TEM (JEM‐1400 Plus, JEOL Ltd., Tokyo, Japan).

### 2.5 Western Blotting Analysis

Samples of cell lysates, sEVs, and m/lEVs derived from Kuramochi cells and samples of sEVs and m/lEVs from ascites and serum were loaded onto polyacrylamide gels and transferred to membranes. After blocking with Blocking One (Nacalai Tesque) for 1 h at room temperature, the membranes were incubated overnight at 4°C with primary antibodies. Anti‐CD9 antibody, clone MM2/57, CBL162 (Merck, Darmstadt, Germany), CD63 antibody (C‐term) EXOAB‐CD63A‐1 (System Biosciences, Palo Alto, CA, USA), and CD81 antibody (B‐11) sc‐166029 (Santa Cruz Biotechnology, Dallas, TX, USA) were used for primary antibodies. Anti‐CD9 and CD81 antibodies were diluted 1:100 in 10% Blocking One/Tris‐buffered saline with 0.1% Tween 20 (TBST), and the CD63 antibody was diluted 1:1,000. The following day, the membranes were washed thrice for 5 min in TBST and incubated for 4 h at room temperature with the secondary antibodies. Anti‐mouse IgG, horseradish peroxidase (HRP)‐linked whole Ab sheep NA931 (Cytiva, Tokyo, Japan) was used for CD9 and CD81 and diluted 1:2000. Anti‐rabbit IgG, HRP‐linked whole Ab donkey NA934 (Cytiva) was used for CD63 and diluted 1:5,000. The membranes were imaged using ImageQuant LAS 4010 (GE Healthcare, Atlanta, GA, USA). Western blotting was performed in two independent experiments, and representative images are shown.

### 2.6 DNA Extraction

Total DNA was extracted from frozen tumor tissue and EVs using DNeasy Blood & Tissue Kits (Qiagen, Hilden, Germany) according to the manufacturer's protocols. DNA was extracted from FFPE tissue at the time of surgery using the QIAamp DNA FFPE Tissue Kit (Qiagen) according to the manufacturer's protocols. The concentration of extracted DNA was quantified using a Qubit 4.0 fluorometer (Invitrogen, Waltham, MA, USA).

### 2.7 Whole‐Genome Sequencing (WGS)

WGS was performed by Novogene company (Beijing, China) using Illumina NovoSeq 6000 (Illumina, San Diego, CA, USA). The genomic DNA was randomly sheared into shorter fragments. The obtained fragments were then end‐repaired, A‐tailed, and further ligated with Illumina adapters. The resulting fragments with adapters were size selected, and PCR amplified unless otherwise specified as PCR‐free, before proceeding for purification. The library was quantified through Qubit and qPCR, and size distribution was detected with a fragment analyzer. Quantified libraries were pooled and sequenced on Illumina platforms according to the effective library concentration and required data amount.

### 2.8 Single Nucleotide Polymorphism (SNP) Array

SNP array analysis was conducted by Riken Genesis (Tokyo, Japan) using the Infinium Asian Screening Array (Illumina). DNA samples were analyzed using the Infinium Asian Screening Array‐24 version 1.0 BeadChips Kit (Illumina). DNA samples were denatured and neutralized by alkali. The denatured samples were amplified by whole‐genome amplification (37°C overnight). Amplified DNA samples were enzymatically fragmented for 1 h at 37°C in a microsample incubator. 2‐Propanol was added to the fragmented DNA samples and precipitated by centrifugation. Precipitated DNA samples were resuspended with a hybridization buffer and incubated for 1 h at 48°C in a hybridization oven. Fragmented and resuspended DNA samples were denatured for 20 min at 95°C in a microsample incubator. Denatured DNA samples were dispensed onto BeadChips using the TECAN system. BeadChips were incubated overnight at 48°C in a hybridization oven to hybridize the samples onto the BeadChips. After hybridization, seals were removed from the hybridized BeadChips. The unhybridized fragment DNA was washed away. Labeled nucleotides were added to the washed BeadChips to extend the primers that hybridized to the DNA. BeadChips were stained, coated for protection, and dried. Dried BeadChips were scanned by the iSCAN system.

SNP genotyping calling and quality control for samples and SNPs were performed using Illumina GenomeStudio v2013 and a cluster file (ASA‐24v1‐0_A1_ClusterFile.egt). Genotypes were scored with GenomeStudio using a GenCall threshold of 0.15. Samples were accepted when their call rates were >96%. SNPs were excluded if they fit the following QC criteria: (a) R mean values of at least one of three clusters were <0.25, (b) Cluster Sep values were <0.4, or (c) the number of no calls value was >2 on all chromosomes, except for the Y chromosome.

### 2.9 Droplet Digital PCR (ddPCR)

CNV of DNA extracted from tissue samples, cell lines, and EVs was determined using a QX200 Auto DG ddPCR system according to the manufacturer's instructions (Bio‐Rad, Hercules, CA, USA). A 22 µL reaction mix containing 11 µL of 2× ddPCR Supermix for Probes (Bio‐Rad), 1.1 µL target probe labeled with a FAM fluorophore, 1.1 µL reference probe labeled with a HEX fluorophore, 1 µL restriction enzyme, and 5 µL DNA template and nuclease‐free water was used for the ddPCR assay. The *RPP30* probe for the ddPCR copy number assay was used as a reference gene. *Mse*I was used as a restriction enzyme for *ERBB2*, and *Hae*III was used for the other target genes. *Mse*I and *Hae*III were diluted fivefold using cut smart diluted 10‐fold with nuclease‐free water. PCR was carried out using the following cycling conditions: 95°C for 10 min, 40 cycles of (94°C for 30 s, 60°C for 1 min), 98°C for 10 min, and 12°C hold. The plates were read on a Bio‐Rad QX200 droplet reader and analyzed using Quanta Soft (Bio‐Rad). For ddPCR analysis, DNA derived from cell lines and tumor tissues was diluted to 0.5–1 ng/µL, and 5 µL was used per reaction. In contrast, EV‐derived DNA was used without dilution due to its low concentration, typically ranging from 0.005–0.05 ng/µL, with 5 µL input per reaction. For cell line experiments, ddPCR analyses were performed in two independent experiments. For clinical samples, due to limited sample availability, ddPCR measurements were performed once for each sample.

The heatmap matrix was made using Morpheus (https://software.broadinstitute.org/morpheus.

### 2.10 Preparation of Polyketone Nanoheterostructures (NHS)‐Coated Nanowires (pNWs) With Antibodies

For synthesizing ZnO NWs, a positive photoresist (OFPR8600, Tokyo Ohka Kogyo Co. Ltd.) was coated on a cleaned glass slide (Matrunami Glass Industry Ltd.) using a piranha solution (1:4 of H2O2, Wako Pure Chemical Industries Ltd., and H2SO4, Wako Pure Chemical Industries Ltd.) at 180°C for 2 h. The coated substrate was exposed to 200 mJ/cm^2^ UV radiation through a channel photomask. The pattern length and width were 19 and 2.5 mm, respectively. Then, the exposed substrate was developed using NMD‐3 2.38 % (Tokyo Ohko Kogyo Co., Ltd.). The ZnO seed layer was deposited using an RF sputter (Sanyu Electron Co., Ltd.). The growth of ZnO NWs was performed by immersing the substrate in a mixture of 50 mM hexamethylenetetramine (Wako Pure Chemical Industries Ltd., Japan) and 50 mM zinc nitrate hexahydrate (Thermo Fisher Scientific) at 95°C for 3 h. After the growth of ZnO NWs, the photoresist was lifted off using acetone and cleaned using ultrapure water. Then, for polyketone coating, a 3 mM polyketoneNHS solution was prepared using chloroform (Fujifilm Wako Pure Chemical Corporation, Japan). 25 µL polyketone solution was dropped onto ZnO NWs and left at room temperature until all solvents had evaporated, and the NWs were completely dried. Three antibodies (FRα, Claudin‐3, TACSTD2) were attached to pNWs by applying 100 µg/mL of each antibody and incubating overnight at room temperature.

### 2.11 Computational Method

All calculations were done with the VASP package and were based on projector‐augmented wave pseudopotentials (Kresse and Furthmuller 1996). Unless otherwise mentioned, the applied density functional was the Perdew‐Burke‐Ernzerhof generalized gradient approximation of exchange‐correlation functional. The cutoff for the plane‐wave basis was 400 eV. The optimized structure of a polyketone chain on a ZnO surface was obtained according to the following steps: (i) A thin ZnO slab, including 360 Zn and 360 O atoms, was prepared by cutting a bulk hexagonal wurtzite crystal structure according to the (1–10) plane. The surface area of the Zn_360_O_360_ slab was ∼2.6 × 2.6 nm, and its thickness was ∼1.1 nm. (ii) The polyketone was attached to the M‐face surface of the Zn_360_O_360_ slab through two adjacent carbonyl groups (O_1_O_2_ coordination) or all four carbonyl groups (O_1_O_2_O_3_O_4_ coordination). (iii) Polyketone‐Zn_360_O_360_ was placed in a periodic cell with a vacuum space of ∼1.2 nm above the polyketone. The size of the final cells was 2.6 × 2.6 × 3.0 nm. (iv) Polyketone‐Zn_360_O_360_ was optimized using the closed‐shell singlet constraint on electronic configuration. The three‐dimensional coordinates of Zn and O atoms in the bottom ZnO layer were fixed during structural optimization, whereas all other atoms were optimized free of constraint. Vibrational frequencies and directions of the four carbonyl groups in the polyketone were calculated using the Hessian matrix and visualized by Jmol. Energies and orbitals of the optimized polyketone‐ Zn_360_O_360_ were calculated with the Heyd‐Scuseria‐Ernzerhof 06 functional. The calculated orbitals were visualized by the VESTA program (Momma and Izumi 2011), with an isovalue of 2.5 × 10^−4^.

### 2.12 sEV Capture by pNWs

sEV extracted from serum was used. After treating the herringbone‐structured polydimethylsiloxane substrate surface with a plasma etching apparatus (Meiwafosis Co., Ltd.), the herringbone‐structured polydimethylsiloxane substrate with microchannels was bonded to the growth NW substrate. The microchannel size was associated with the NW pattern. Poly ether ether ketone (PEEK) tubes were then connected to the herringbone‐structured polydimethylsiloxane substrate and to a syringe via a fluoropolymer connector (Institute of Microchemical Technology Co. Ltd.), and serum sEVs were supplied using a syringe pump. Then, 250 µL of the sEVs were applied into the channel at 50 µL/min to trap EVs on the pNWs with antibodies. Last, 150 µL of Dulbecco's PBS (Nacalai Tesque) was injected at 50 µL/min to collect EVs trapped on the pNWs with antibodies.

### 2.13 Predictive Model for PARP Inhibitor Response

PCA of the CNV status was performed using the prcomp and plot3d functions of the rgl package (version 0.100.54). The cross‐validation (CV) score was calculated for each selected gene to evaluate the contribution of PARP inhibitor response. The CV score indicates the robustness of discrimination performance between two groups based on Fisher's linear discriminant analysis (LDA) with leave‐on‐out CV. The 30 genes were ranked based on CV score, and a CV score >0.75 was judged as significant and contributing to the prediction of PARP inhibitor response.

The sensitivity, specificity, and accuracy of the panel for ddPCR analysis in predicting response to PARP inhibitor treatment were determined using receiver operating characteristic (ROC) curve analysis, and the area under the curve (AUC) was calculated. To calculate sensitivity, specificity, and accuracy, optimal cutoff values were set based on the maximum point of the sum of sensitivity and specificity (i.e., the Youden index). The 95% confidence interval (95% CI) of the AUC was calculated and plotted in the ROC curve. The best combination model for predicting response to olaparib was developed by logistic least absolute shrinkage and selection operator (LASSO) regression analysis. Fisher's LDA and logistic LASSO regression analysis were performed using R version 4.3.0 (R Foundation for Statistical Computing; http://www.R‐project.org), compute.es (version 0.2–4), glmnet (version 2.0–3), hash (version 2.2.6), MASS (version 7.3–45), mutoss (version 0.1–10), and pROC (version 1.8).

### 2.14 Cell Viability Assay

For the multicellular tumor spheroid assay, cells were seeded into a 96‐well plate and cultured for 24 h. Cell viability assays were performed in two independent experiments, each with triplicate wells per condition, and representative results are shown. The medium was replaced with an olaparib (MedChemExpress, Monmouth Junction, NJ, USA)‐containing medium, and cells were incubated for 48 h. Finally, 10 µL of 5 mg/mL CellTiter 96 Aqueous One Solution (Promega, Madison, WI, USA) were added to each well and incubated with cells for 3 h. Optical density was determined using a spectrophotometer at a wavelength of 490 nm. Cell viability was calculated as (OD_cisplatin_ − OD_blank_)/(OD_control_ − OD_blank_) × 100 using GraphPad Prism version 10.0. The IC_50_ value was calculated using the following equation: IC_50_ (µM)  =  10[log(A/B) × (50−C)/(D−C) + log(B)], where A and B represent the highest and the lowest concentrations (µM) to cover an estimated IC_50_ value, respectively. C and D represent the cell viability at concentrations B and D, respectively.

### 2.15 Statistical Analysis

All statistical analyses were performed using GraphPad Prism version 10.0 (GraphPad Software, San Diego, CA, USA) and R version 4.3.0 (R Foundation for Statistical Computing, Vienna, Austria). Continuous variables between two groups were compared using the Mann–Whitney U test. Comparisons among multiple groups were performed using the Kruskal–Wallis test followed by Dunn's multiple comparison test. Correlations were assessed by linear regression analysis, and R^2^ indicates the coefficient of determination. Receiver operating characteristic (ROC) curve analysis was used to evaluate predictive performance, and the area under the curve (AUC) was calculated. Statistical significance was defined as *p* < 0.05.

## 3 Results

### 3.1 Isolation of sEVs and M/lEVs

**FIGURE 1:**
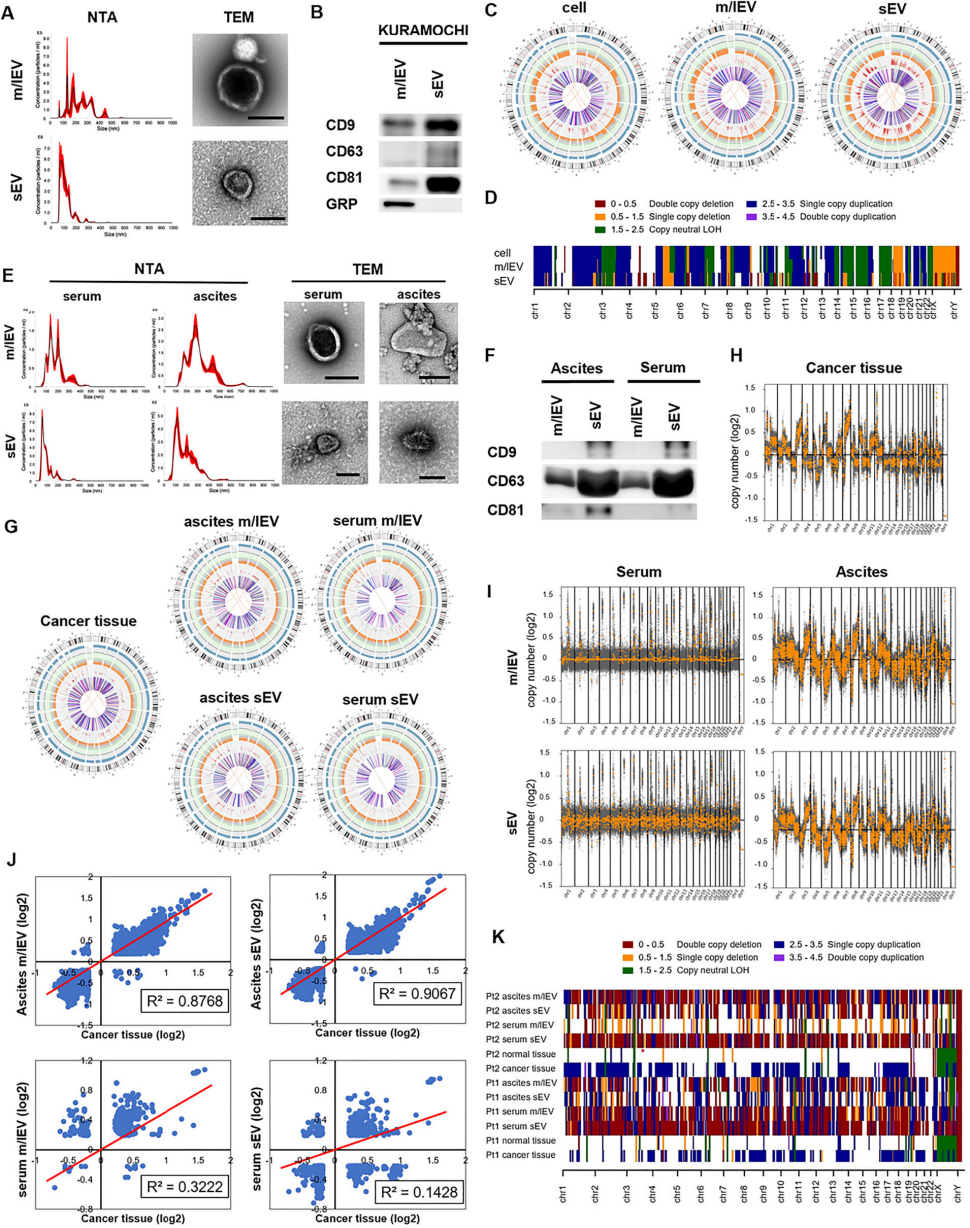
Presence of CNV information in EV‐DNA. (a) Nanoparticle tracking analysis of sEVs and m/lEVs for Kuramochi cells and representative TEM images from EVs. Scale bar, 200 nm (m/lEVs) and 100 nm (sEVs). (b) Western blotting analysis of CD9, CD63, CD81, and GRP for sEVs and m/lEVs from Kuramochi cells. (c) Circos plots of WGS from Kuramochi cells and EVs. (d) Heatmap of the SNP array from Kuramochi cells and EVs. (e) Nanoparticle tracking analysis of sEVs and m/lEVs for a patient derived from HGSOC and representative TEM images from EVs. Scale bar, 200 nm for m/lEVs and 100 nm for sEVs. Nanoparticle tracking analysis was performed in duplicate for each sample, and representative results are shown. (f) Western blotting analysis of CD9, CD63, and CD81 for sEVs and m/lEVs from a patient with HGSOC. (g) Circos plots of WGS from a patient with HGSOC. (h and (i) CNV status in cancer tissue and ascites‐derived EV‐DNA from a patient with HGSOC. Profiles demonstrate somatic chromosomal gains (top) and losses (bottom). (j) Correlations of CNV status between cancer tissue and EVs of serum or ascites. R2 is the determination coefficient. (k) Heatmap of the SNP array from patients with HGSOC. Western blotting was performed in two independent experiments, and representative images are shown.

EVs were isolated according to the standard differential centrifuge method for cell lines and clinical samples. It was confirmed that EVs were properly collected by TEM, and nanoparticle tracking analysis was performed to assess the size distribution of each type of EV (Figure 1a and e). To examine EV marker protein levels, Western blotting analysis was performed for the conventional EV markers CD9, CD63, and CD81 and the non‐EV marker GRP. For cell lines, CD9, CD63, and CD81 were positive in sEVs and m/l EVs, whereas only GRP was positive in m/lEVs (Figure 1b). For clinical samples, CD9, CD63, and CD81 were positive in sEVs, whereas only CD63 was clearly positive in m/lEVs (Figure 1f). Uncropped Western blot images corresponding to Figure 1b and Figure 1f are provided in Figure S1.

### 3.2 CNV Analysis by WGS and SNP Array

To analyze the comprehensive CNV status, WGS and SNP array were performed. The circos plot of the WGS showed that a similar tendency of CNVs was observed in cells and EVs (Figure 1c). The heatmap for the SNP array also showed similar CNV profiles in cells and EVs (Figure 1d). For clinical samples with HGSOC, collected at the time of primary debulking surgery, WGS showed that the CNV status of EV‐DNA in ascites (ascEV‐DNA) showed concordance with the status of tumor‐DNA (Figures 1h–j), whereas the status of EV‐DNA in serum (serEV‐DNA) did not reflect that of tumor DNA probably because the concentration of DNA derived from the tumor was very low. However, the SNP array did not show an association of CNVs between the tumor tissue and ascites/serum EVs (Figure 1k). This could be because there was not enough DNA in EVs for SNP analysis and the presence of other blood cells or interstitial components may have affected the analysis.

### 3.3 Gene Selection for ddPCR

**FIGURE 2:**
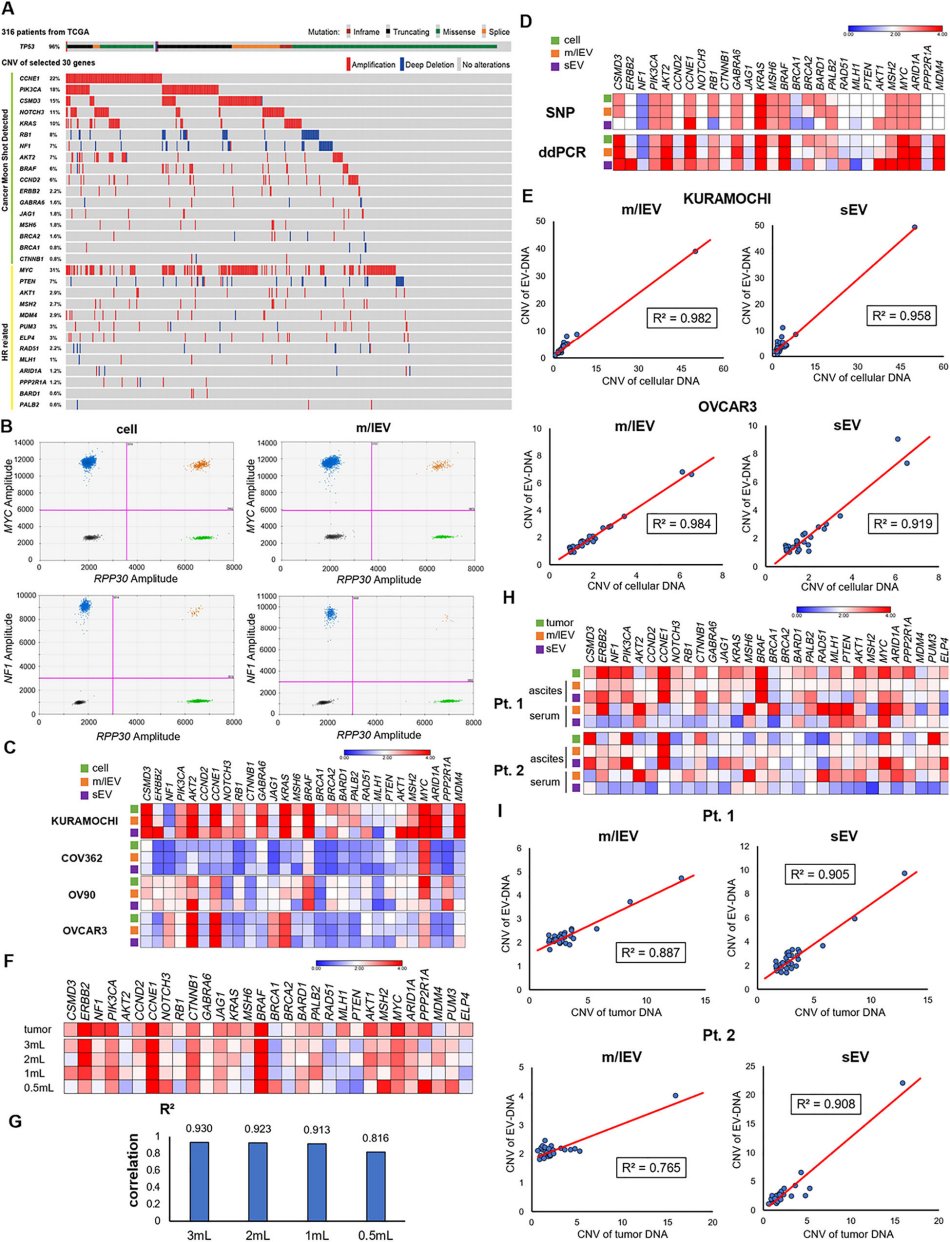
Target CNV in EV‐DNA evaluation by ddPCR. (a) Proportion of gene alterations from TCGA for the selected genes for analysis by ddPCR. Seventeen genes were selected by referring to the OCMS database, whereas 13 genes were selected as HR‐related genes. (b) Representative two‐dimensional (2D) amplitude of ddPCR for Kuramochi cells. Blue means positive for the target gene, green means positive for the housekeeping gene, orange means double positive, and gray means double negative. RPP30 was used as a housekeeping gene. (c) Matrix of CNVs for cell lines and EVs. These four cell lines were derived from HGSOC. The color scale represents absolute copy number values, where 2 corresponds to copy number neutral. Values greater than 2 are shown in red (copy number gain), whereas values less than 2 are shown in blue (copy number loss). (d) CNV status of the SNP array and ddPCR focusing on the 30 selected genes for Kuramochi cells and EVs. (e) Correlations of CNV status between cellular DNA and EV‐DNA for Kuramochi and OVCAR3 cells. (f) CNV status of EV‐DNA extracted from ascites from different volumes and the status of the original tumor. (g) R2 values with CNVs of tumor DNA depending on the starting amount of ascites. (h) Matrix of CNV status for the tumor tissue and EVs from two patients with HGSOC. (i) Correlations of CNV status between tumor DNA and EV‐DNA in ascites from two patients with HGSOC. ddPCR analyses for cell lines were performed in two independent experiments, whereas clinical samples were analyzed once due to limited sample availability. Correlations were assessed by linear regression, and R2 indicates the coefficient of determination.

Thirty genes were selected as the dominant panel for analysis by ddPCR based on the OCMS database (Lee et al. 2020) and the reports on HR repair (Garsed et al. 2018) and response to the PARP inhibitor (Amuzu et al. 2021). The selected genes were referenced to TCGA in 2011^28^ to confirm that copy number changes actually occurred in HGSOC (Figure 2a).

### 3.4 CNV Analysis by ddPCR

The concept of analyzing CNVs in EVs by WGS was proven, but WGS is not practical in actual clinical settings because WGS analysis is expensive and requires a large amount of DNA. Therefore, selective and sensitive assays are required, and the CNV status of specific genes was analyzed by ddPCR, characterized by high precision and sensitivity. Figure 2b shows the actual ddPCR results for Kuramochi cells. Blue dots mean positive droplets for the target gene, green dots mean positive droplets for the housekeeping gene, orange dots mean double positive, and gray dots mean double negative. The copy number is calculated by referring to the ratio of target to housekeeping gene‐positive droplets. *MYC* amplification and *NF1* loss were detected in EV‐DNA (Figure 2b). CNV analysis was performed using ddPCR on 30 panel genes in cell lines derived from HGSOC. All cell lines showed high correlations of CNV status between cellular DNA and EV‐DNA (Figure 2c). Focusing on the 30 gene panel, the SNP array and ddPCR results were compared. The CNV status of cellular DNA by the two analyses showed similar profiles, and both were detected using EV‐DNA (Figure 2d). The correlations were examined according to the EV size for cell lines. sEVs and m/lEVs from cell lines showed high correlations of CNVs between cellular DNA and EV‐DNA (Figure 2e; Figure S2).

The DNA amount in EVs in body fluids is very small. Therefore, the minimum volume that can be detected by ddPCR was investigated. sEVs from 0.5 to 3 mL of ascites were isolated from a patient with HGSOC at the time of surgery, and CNV analysis was performed using ddPCR (Figure 2f). As a result, the correlation of the CNV status between tumor DNA and ascEV‐DNA decreased with 0.5 mL of ascites (Figure 2g; Figure S3). Therefore, a minimum of 1 mL of fluid was used for analysis in subsequent ddPCR experiments. Next, CNV analysis by ddPCR was performed for clinical samples (Figures 2h and i; Figure S4). EVs were collected from 1 mL of serum or ascites from two patients derived from HGSOC. Similar to WGS results, CNV of ascEV‐DNA showed concordance with the status of tumor DNA (Figure 2h). The correlations according to the EV size for clinical samples were investigated. The CNV status in sEVs in ascites showed higher correlations with the status of tumor DNA than m/lEVs in both cases (Figure 2i), probably due to differences in EV biogenesis, as sEVs are more actively generated than m/lEVs. In summary, the CNV status of cellular or tumor DNA can be detected by analyzing EV‐DNA from cell lines or ascEV‐DNA using ddPCR.

### 3.5 Increased DNA in Ascites is Associated With Malignancy

The clinical applications of this assay were considered. Before evaluating CNV analysis in EVs, how CNVs are altered in ovarian cancer tissue compared to normal ovaries was examined. DNA was extracted from FFPE tissue of normal ovaries from patients with benign ovarian tumors and ovarian cancer tissues, and CNV analysis by ddPCR was performed. Patients’ characteristics are shown in Table S1. As a result, normal ovarian tissue had a unique copy number of each gene in common, and the copy numbers of *RAD51*, *BRCA1*, *AKT2*, *CCNE1*, and *MSH6* were significantly higher in malignant tumors (Figures S5 and S6).

**FIGURE 3:**
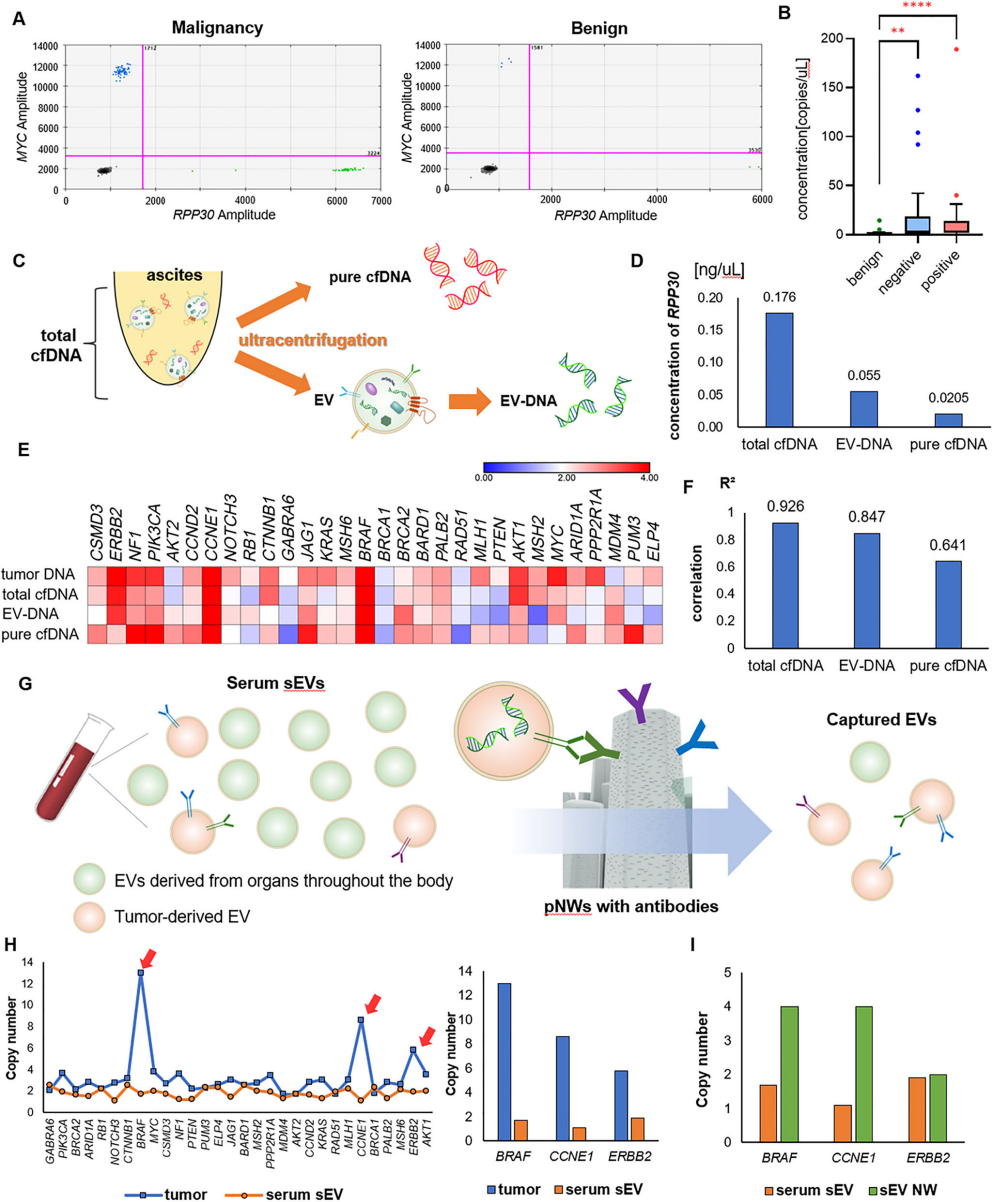
EV‐DNA serves as a useful source of tumor‐associated genomic information. (a) Representative 2D amplitude of ddPCR for malignant and benign ascites. EVs were extracted from 1 mL of undiluted ascites. (b) RPP30 concentrations in EV‐DNA measured by ddPCR. EVs were extracted from patients with benign ovarian tumors (*n* = 26), cytology‐negative ovarian cancer (*n* = 34), and cytology‐positive ovarian cancers (*n* = 36). ** indicates *p* < 0.005; **** indicates *p* < 0.0001. Data were compared using the Kruskal–Wallis test followed by Dunn's multiple comparison test. (c) Schema for comparison of cfDNA and EV‐DNA. Conventional cfDNA was defined as the total cfDNA and DNA obtained from the supernatant as pure cfDNA. (d) RPP30 concentrations measured by ddPCR. (e) Matrix of CNVs measured by ddPCR for tumor DNA, total cfDNA, EV‐DNA, and pure cfDNA from a patient with HGSOC. The color scale represents absolute copy number values, where 2 corresponds to copy number neutral. Values greater than 2 are shown in red (copy number gain), whereas values less than 2 are shown in blue (copy number loss). (f) R2 values representing the correlation between tumor DNA and total cfDNA, EV‐DNA, and pure cfDNA. Correlations were assessed by linear regression, and R2 indicates the coefficient of determination. (g) Schema for capturing EV populations carrying tumor‐associated DNA from serum sEVs using pNWs with antibodies. Serum EVs obtained from patients with HGSOC were passed through pNWs. DNA was collected by passing PBS through NWs. (h) CNV status of tumor DNA and serum sEV‐DNA. The arrows indicate the top 3 most amplified genes in tumor DNA. (i) Copy numbers of BRAF, CCE1, and ERBB2, the top 3 most amplified genes in tumor DNA before and after passing through the pNWs.

CNV evaluation was performed by ddPCR using 1 mL of undiluted ascites, not peritoneal wash, from patients with benign ovarian tumors (*n* = 26) and ovarian cancer (*n* = 68) at the time of surgery. Patients’ characteristics are shown in Table S2. Very little DNA in EVs was present in ascites from benign patients (Figure 3a), and the concentration of *RPP30*, a housekeeping gene, was significantly higher in ascites EVs from cancer patients (Figure 3b; **Kruskal–Wallis test followed by Dunn's multiple comparison test, *p* < 0.0001**), suggesting that increased ascEV‐DNA itself may be associated with the presence of malignancy. Furthermore, even in ovarian cancer patients with negative ascitic cytology, the concentration of ascEV‐DNA was significantly higher than that in benign ascites (*p* = 0.0023), whereas no significant difference was observed between cytology‐negative and cytology‐positive groups (*p* > 0.9999), suggesting that ascEV‐DNA levels may increase from an earlier stage of disease development. Similar results were obtained even after excluding cytology‐suspicious cases from the cytology‐negative group (Figure S7).

### 3.6 EV‐DNA Serves as a Useful Source of Tumor‐Associated Genomic Information

Cell‐free DNA (cfDNA) is present as EV‐DNA or macromolecular complexes in body fluids. In general, cfDNA results from necrotic cells and is unstable, whereas EV‐DNA is more stable (García‐Silva et al. 2020). Our group previously reported on the mechanism of DNA loading into exosomes, that is, cancer cells actively load their own DNA into exosomes via micronuclei (Yokoi et al. 2019), consistent with the results of increased ascEV‐DNA of patients with ovarian cancer. Therefore, it was assumed that cfDNA may be largely associated with EVs. Conventional cfDNA was defined as total cfDNA, DNA obtained from the ultracentrifuge pellet as EV‐DNA, and DNA obtained from the supernatant as pure cfDNA, and these CNVs were measured by ddPCR (Figure 3c). The *RPP30* concentration was higher in EV‐DNA compared to pure cfDNA (Figure 3d). The CNV status of total cfDNA and EV‐DNA showed high correlations with the status of tumor DNA, whereas the status of pure cfDNA showed lower correlations (Figure 3e and f; Figure S8), suggesting that cfDNA derived from tumor cells may be enriched in EV fractions.

### 3.7 Selective Capture of EV Populations Associated With HGSOC Using Antibody‐Coated Nanowires

The problem with CNV analysis is that it is hard to investigate the tumor CNV status in serum EVs due to the dilution of EVs carrying tumor‐associated DNA. As mentioned earlier, EV‐DNA is actively released from cancer cells, suggesting that EV fractions may be enriched for tumor‐derived genomic information. As a feature of EVs, EVs are lipid bilayer structures enriched with membrane proteins (Yokoi and Ochiya 2021), enabling the selective capture of specific EV populations using membrane markers. Our group previously reported that the marker proteins associated with HGSOC‐derived EV populations, including Claudin‐3, folate receptor α (FRα), and tumor‐associated calcium signal transducer 2 (TACSTD2), were identified using polyketone‐coated nanowires (Yokoi et al. 2023). Therefore, whether EV populations enriched for tumor‐associated DNA could be selectively captured from serum EVs using pNWs with antibodies was investigated.

**FIGURE 4:**
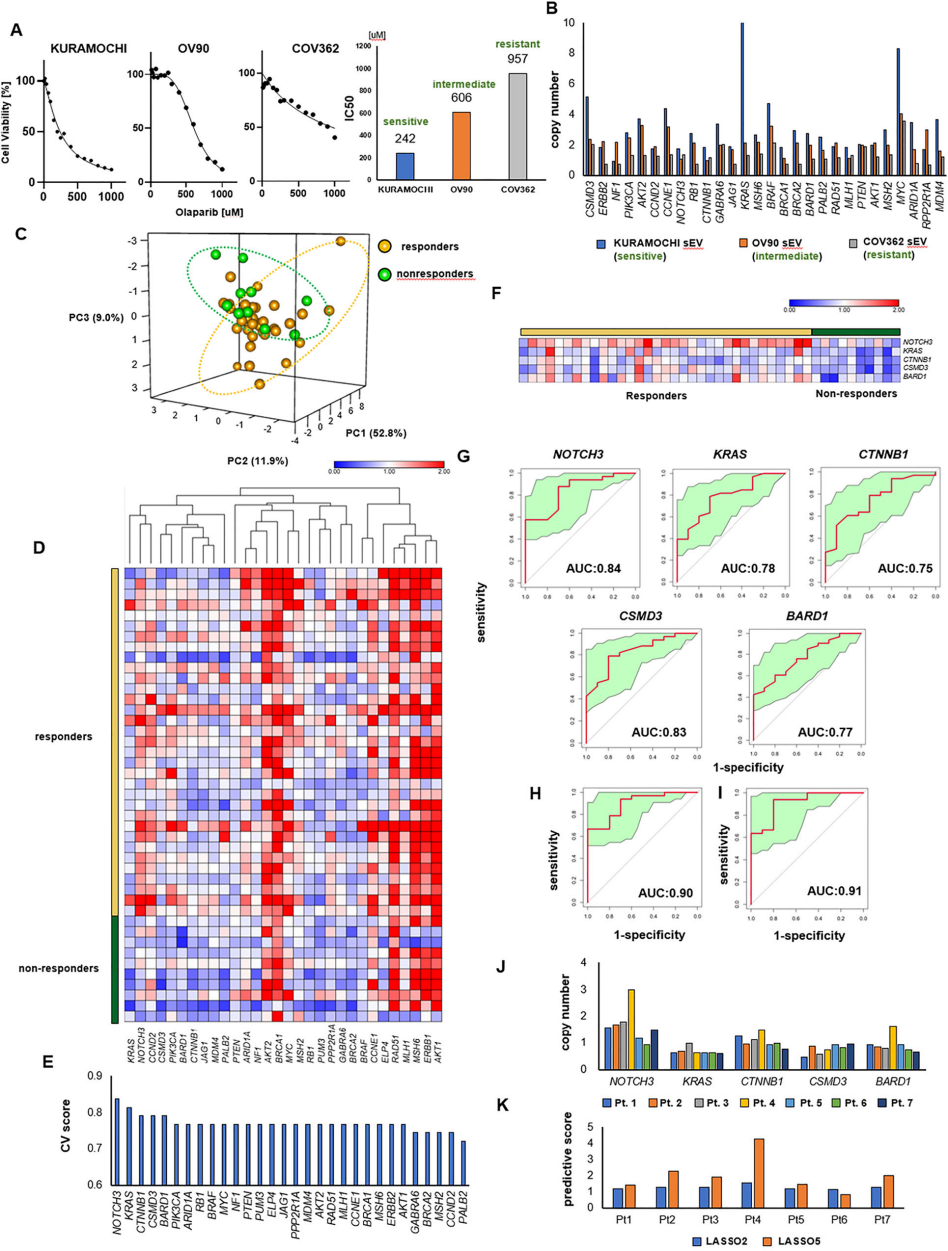
EV‐DNA as companion biomarker for PARP inhibitor treatment. (a) IC50 values of Kuramochi, OV90, and COV362 cells for olaparib. Kuramochi cells were classified as sensitive, OV90 cells as intermediate, and COV362 cells as resistant. (b) CNV status for sEV‐DNA from Kuramochi (sensitive), OV90 (intermediate), and COV362 (resistant) cells. (c) PCA of CNV profiles for responders and nonresponders. (d) Hierarchical clustering and heatmap showing the CNV profiles of responders and nonresponders. The copy number values were normalized to those of normal ovarian tissue derived from FFPE samples, with normal copy number defined as 1. The color scale represents relative copy number values, where values greater than 1 are shown in red (copy number gain), whereas values less than 1 are shown in blue (copy number loss). (e) CV scores showing the predictive performance of the 30‐gene panel. (f) Heatmap for the top 5 genes of CV scores. (g–i) ROC curves and AUC values for the selected gene panels. (g) Top 5 genes ranked by CV score. (h) Combination of NOTCH3 and CSMD3 using LASSO analysis. (i) Combination of ARID1A, NOTCH3, CSMD3, ELP4, and BARD1 using LASSO analysis. The green area represents the 95% confidence interval (CI), and AUC values are shown with corresponding CIs. (j) Copy numbers of the top 5 genes of CV scores for sEV‐DNA in ascites from patients with HGSOC who received olaparib as maintenance therapy. (k) Predictive scores of LASSO2 and LASSO5 for patients with HGSOC who received olaparib as maintenance therapy. The respective prediction equations were LASSO2: 0.2133 × NOTCH3 + 0.1967 × CSMD3 + 0.7894 and LASSO5: 0.24758 × ARID1A + 1.60957 × NOTCH3 + 1.95855 × CSMD3 + 0.02955 × ELP4 + 0.11231 × BARD1 – 2.6119. When the value of the equation is >0, the patient's prognosis is considered good; when it is <0, the prognosis is considered poor.

Claudin‐3, FRα, and TACSTD2 antibodies were modified to pNWs. Serum EVs obtained from a patient with HGSOC were passed through pNWs, DNA was extracted from captured EVs, and CNV analysis was performed by ddPCR (Figure 3g). Figure 3h shows the CNV status of tumor DNA and serum sEVs. Focusing on the three most amplified genes in the tumor tissue, the copy number was elevated in two of three genes after passing through pNWs with antibodies (Figure 4i), indicating that specific EVs could be captured on the pNWs with antibodies. Similar analyses were performed in two additional cases, in which the most amplified genes could be successfully identified after passing through pNWs (Figure S9).

### 3.8 CNV Status Could Predict Response to PARP Inhibitor Treatment

The potential of CNV status as a prognostic biomarker was examined next. IC_50_ values to olaparib for three different cell lines derived from HGSOC were measured and classified as sensitive, intermediate, or resistant according to their sensitivity to olaparib (Figure 4a). In this IC_50_ assay, we used cell lines such as KURAMOCHI, OV90, and COV362, which are representative of HGSOC cell lines (Domcke et al. 2013). The CNV status of EV‐DNA for these cell lines was measured, and higher sensitivity to olaparib was associated with higher copy numbers (Figure 4b). Therefore, it was assumed that CNV status and sensitivity to olaparib were correlated, and the relationship between CNV status and response to olaparib treatment was investigated.

Patients with HGSOC who were treated with olaparib as maintenance therapy were included in this study. Responders were defined as patients who received olaparib continuously for >6 months (*n* = 33), whereas nonresponders were those who discontinued the drug within 6 months due to recurrence or disease progression (*n* = 9). Patients’ characteristics are shown in Table 1. The responders included BRCA‐negative patients, and both groups included chemotherapy naïve and postchemotherapy samples. DNA was extracted from FFPE tissue at the time of surgery, and CNV analysis was performed by ddPCR. Principal component analysis (PCA) and the heatmap showed that CNV profiles differed between responders and nonresponders (Figure 4c and d). A heatmap showing the CNV profiles before normalization is presented in Figure S10. In terms of the timing of sample collection, 52% of the responders and 56% of the nonresponders were chemo‐naïve patients. When analyzed separately according to chemotherapy history, responders consistently demonstrated higher copy number levels than nonresponders in both chemotherapy‐naïve and previously treated groups (Figure S11). The predictive performance of each gene was evaluated using the CV score, and the top 5 genes were selected as candidates for predicting response (Figure 4e). The profiles of the heatmap for the selected genes were different, and responders showed higher copy numbers (Figure 4f). The ROC curves for each gene showed a good AUC values (Figure 4g). Furthermore, a combination of two genes, NOTCH3 and CSMD3, resulted improved predictive performance (AUC = 0.90) (Figure 4h). Adding three additional genes, *ARID1A*, *ELP4*, and *BARD1*, resulted in an AUC of 0.91 (Figure 4i). Thus, CNV analysis could serve as an alternative predictive biomarker for response to PARP inhibitors.

The potential of the selected genes was validated using EV‐DNA. AscEV‐DNA at the time of surgery was collected from seven patients who received maintenance therapy with olaparib. Patient characteristics are shown in Table 2. The copy numbers of ascEV‐DNA tended to show higher values in cases with longer treatment durations (Figure 4j). The prediction equation based on the two and five genes showed consistent classification of treatment response, although all seven patients were responders who had continued the treatment for more than six months (Figure 4k). In this way, the selected genes were validated using ascEV‐DNA from clinical samples.

**TABLE 2:** Patient characteristics for CNV analysis from EV‐DNA in ascites treated with olaparib for maintenance therapy.

Pt. number	Age	Duration(days)	Sample collection	FIGO stage (2014)	Somatic *BRCA* mutation	HRD
1	47	46	PDS	IIIA	*BRCA2* Positive	—
2	33	24	Exploratory laparotomy	IIIB	BRCA1 positive	—
3	41	24	Exploratory laparotomy	IIIC	Negative	Positive
4	71	11	PDS	IIIC	Negative	—
5	51	11	Exploratory laparotomy	IIIC	*BRCA1* positive	Positive
6	43	8	IDS	IVA	Negative	Negative
7	63	8	IDS	IIIC	Negative	—

Abbreviations: HGSOC, high‐grade serous ovarian carcinoma; HRD, homologous recombination deficiency; PDS, primary debulking surgery.

## 4 Discussion

This study highlighted the potential of EV‐DNA as a biomarker in diagnosing and predicting treatment response for HGSOC. CNV profiles in ascEV‐DNA closely reflected the genomic status of tumor DNA but not those in serum‐derived EV‐DNA. Ascites exists in the same abdominal cavity as the ovarian tumor and are in direct contact with it. Cancer cells actively release DNA into EVs^22^, suggesting that EVs closely reflecting the tumor genomic status are enriched in ascites. In contrast, serEV‐DNA did not correlate as strongly with tumor DNA as ascEV‐DNA, probably because EVs from multiple tissues circulate in the bloodstream, where EVs carrying tumor‐associated DNA are relatively diluted.

One of the key findings of this study is that ascEV‐DNA in patients with HGSOC reflected the CNV profile of tumor DNA more closely than cfDNA that excluded EVs, and EV‐DNA contained a higher amount of DNA than cfDNA, suggesting that a substantial proportion of cfDNA may exist in association with EVs. One study reported that cfDNA also exists mainly in EVs in the blood (Fernando et al. 2017), and this study might support this finding. As cfDNA is thought to reflect the information from apoptotic or necrotic cells and EVs form complexes (García‐Silva et al. 2020), EV‐DNA may show greater stability and improved retention of tumor‐associated genomic information, making it a promising resource for liquid biopsy. As useful applications of cfDNA in liquid biopsy, one study reported that targeted therapy based on circulating tumor DNA (ctDNA) profiling demonstrated significantly improved overall survival (Nakamura et al. 2024). Furthermore, the concept of minimal residual disease is also known as a poor prognostic factor in various carcinomas, where ctDNA detection is after standard treatment (Pantel and Alix‐Panabieres 2019). EV‐DNA may be a potentially better alternative material for these liquid biopsies. There is still no definitive consensus on the mechanism of EV‐DNA biosynthesis or its location. Our group previously reported on the mechanism of DNA loading into exosomes that tumor cells actively release their own DNA into exosomes via micronuclei (Yokoi et al. 2019), whereas another study reported that DNA inclusion in tumor‐derived microvesicles is associated with cGAS but not amphisome or micronuclei components (Clancy et al. 2022). Some EV studies reported that DNA existed inside the EV lumen as DNA was protected from nuclease degradation (Clancy et al. 2022; Fernando et al. 2017), whereas other studies suggested that most DNA was associated with the EV surface (Lázaro‐Ibáñez et al. 2019; Liu et al. 2022). This may also reflect differences in EV biogenesis and cargo loading between EV subtypes, as previous studies have suggested that distinct EV populations can differ in their molecular contents, including nucleic acids, depending on their biogenesis pathways (Colombo et al. 2014). Mechanistically, sEVs are generally generated via the endosomal pathway, whereas m/lEVs are formed through direct budding from the plasma membrane, which may lead to differences in membrane protein composition and EV‐DNA content (Akers et al. 2013), thereby influencing CNV profiles across EV subtypes. Consistently, heterogeneity in EV marker expression has been widely reported in clinical samples (Wiklander et al. 2015, Kowal et al. 2016), reflecting the diverse cellular origins and biological characteristics of EV populations. In our study, EV characteristics were further confirmed using nanoparticle tracking analysis and transmission electron microscopy, supporting the presence of heterogeneous EV populations. Ascites is a complex biofluid containing EVs derived not only from tumor cells but also from various components of the tumor microenvironment, including stromal, mesothelial, endothelial, and immune cells (Yáñez‐Mó et al. 2015, Lengyel 2010). Therefore, the EV‐DNA detected in ascites likely represents a composite genomic signal rather than exclusively tumor‐derived DNA. At the same time, it is noteworthy that EV‐DNA profiles in ascites closely resemble tumor genomic profiles despite this heterogeneity. One possible explanation is that cancer cells may be more likely to release EV‐associated DNA, as suggested in our previous study (Yokoi et al. 2019). In addition, the detection of EV‐DNA even in cytology‐negative ascites in our cohort is consistent with this possibility and supports the contribution of tumor‐derived DNA to the overall EV‐DNA signal.

Although our results suggest that ascites‐derived EV‐DNA is enriched for tumor‐associated genomic alterations compared to serum, this does not imply tumor specificity. Future studies incorporating tumor‐specific EV enrichment strategies will be important to further improve specificity.

Compared to benign cases, the increased ascEV‐DNA associated with malignant cases suggested that ascEV‐DNA could serve as a marker for malignancy. Our group previously reported that tumor cells actively load their own DNA into exosomes via micronuclei, (Yokoi et al. 2019) consistent with the increased ascEV‐DNA levels observed in ovarian cancer patients. Furthermore, the finding that elevated ascEV‐DNA can be detected even in cytology‐negative cases implied that ascEV‐DNA may serve as a potential marker for early detection, potentially identifying malignancy before cytological abnormalities appear. This early detection is crucial in ovarian cancer, where symptoms often remain subtle until the disease progresses to advanced stages.

The ability of CNV profiling to predict responses to PARP inhibitors represents a promising advance in personalized medicine for ovarian cancer. This study identified a five‐gene CNV signature (*ARID1A*, *NOTCH3*, *CSMD3*, *ELP4*, and *BARD1*) from FFPE tissue with an AUC value of 0.91 for predicting olaparib response. This gene panel could be useful in selecting patients likely to benefit from PARP inhibitor therapies. This approach may address unmet clinical needs, as current biomarkers for PARP inhibitors, including *BRCA* mutations and HR status, do not consistently predict patient outcomes. This panel correctly differentiated responders from nonresponders, including a patient with an HRD tumor who developed treatment resistance. The CNV‐based gene panel may reflect the tumor's genetic alterations, potentially providing accurate information on tumor susceptibility to PARP inhibitors and allowing clinicians to choose therapies accordingly.

Previous studies on EV‐based biomarkers in ovarian cancer have predominantly focused on microRNAs or proteins (Chang et al. 2019, Giannopoulou et al. 2019). In a previous report on biomarkers using CNVs in ovarian cancer, CNV detection of ctDNA in serum by shallow WGS was useful for monitoring disease evolution in HGSOC (Paracchini et al. 2021). WGS is not a practical or cost‐effective approach in clinical settings; therefore, a targeted PCR‐based method was adopted, and candidate genes were selected for ddPCR analysis. This study demonstrates the potential of CNVs in EV‐DNA as biomarkers in HGSOC. The study results aligned with growing evidence that CNV profiles in tumors play a critical role in ovarian cancer pathogenesis and therapeutic response (Cheng et al. 2022; Macintyre et al. 2018; Martins et al. 2022; Smith et al. 2023). Beyond ovarian cancer, this approach could be adapted for other malignancies where CNVs play a significant role in disease progression and therapeutic response. The methodology described here, particularly ddPCR for detecting CNVs in EV‐DNA, may be useful for liquid biopsy applications in cancers with high CNV prevalence. Importantly, unlike comprehensive sequencing‐based approaches, this study employed a targeted ddPCR platform, which enables practical, cost‐effective, and reproducible assessment of CNV profiles using a limited number of genes. This feature may support the clinical use of EV‐DNA–based biomarkers. However, prospective clinical studies will be required to validate the clinical utility of this approach as a biomarker.

In clinical practice, we often encounter patients with poor general condition who are not suitable candidates for surgery. In such cases, tissue sampling is often difficult, and diagnosis or molecular profiling becomes challenging. However, ascitic fluid can be safely accessed even in patients who are not surgical candidates, for example, through the Douglas pouch puncture. Therefore, analyzing EV‐DNA obtained from ascites could provide clinically meaningful genomic information without requiring invasive procedures. The ability to predict therapeutic response or obtain supplementary diagnostic information from EV‐DNA profiles in ascites could be particularly valuable for patients who cannot undergo surgery or tissue biopsy. This approach would expand the clinical utility of EV‐DNA analysis beyond advanced cases, potentially contributing to earlier and more individualized treatment decisions. Moreover, our study is not simply using EVs as a proxy for malignancy. Rather, we show that ascites‐derived EV‐DNA contains richer biological information that reflects patient‐specific tumor characteristics and potential treatment responsiveness. Importantly, ascites has had no established clinical use beyond determining the presence or absence of malignancy and assessing cytology. Our findings indicate that ascites EV‐DNA can provide deeper molecular insights, thereby expanding the clinical utility of ascites beyond its traditional role. However, ascites is typically available in advanced‐stage disease, and its applicability may be limited in early‐stage patients or in cases where ascites is not accessible. At the same time, ascites can also be present in a subset of early‐stage patients, suggesting that this approach may still be applicable in selected cases. Importantly, recent advances in EV collection technologies have enabled the recovery of EVs even from samples without apparent ascites, such as through EV sheet–based approaches (Yokoi et al. 2023), indicating that EV‐DNA–based analyses may be extended to earlier‐stage disease settings in the future.

Although the findings of this study are promising, there are certain limitations. First, the sample size for patient‐derived EV‐DNA was relatively small, particularly for patients receiving PARP inhibitor therapy. Larger studies with diverse cohorts must validate these findings and ensure that the proposed CNV signature is broadly applicable. Another limitation of this study is the use of serum rather than plasma for EV isolation. While plasma is sometimes preferred to minimize platelet‐derived vesicle contamination, serum is also commonly used in many institutional and national biobanks, and remains an acceptable source for EV studies according to the latest MISEV2023 guidelines (Lucien et al. 2023; Nieuwland and Siljander 2024). In addition, EVs isolated from serum may include a substantial proportion of platelet‐derived vesicles, which are known to be abundant in serum due to the coagulation process (Zhang et al. 2022). This may influence EV‐DNA measurements and contribute to the lower concordance observed between serum EV‐DNA and tumor DNA in our study. Importantly, the DNA extracted from serum EVs in this study was sufficient for both WGS and digital PCR, suggesting that the use of serum did not critically affect data quality. Furthermore, this study reported that serEV‐DNA did not correlate as strongly with tumor DNA as ascEV‐DNA. This may be due to the low abundance of EVs carrying tumor‐associated DNA in serum. Further validation in larger cohorts is required.

In addition, ddPCR is a highly sensitive method capable of detecting low‐abundance DNA. However, its quantitative precision may be affected under low DNA input conditions. This is because ddPCR relies on Poisson‐based partitioning, which can introduce variability when DNA copy numbers are low (Hindson et al. 2011; Milbury et al. 2014). Therefore, careful interpretation of ddPCR‐based CNV data is required, particularly when analyzing EV‐derived DNA from clinical samples with limited DNA availability. To overcome this issue, future research should focus on developing methods for enriching EV populations carrying tumor‐associated DNA in serum, including capturing techniques such as pNWs that selectively isolate EV populations associated with HGSOC. Although this study found the potential of pNWs, the number of samples used was limited, and further investigation is needed. Although the number of patients analyzed in this study was limited, it is noteworthy that several responders to PARP inhibitors were *BRCA*‐negative, who are generally considered less likely to benefit from these agents. The ability to identify potential responders even among *BRCA*‐wildtype patients suggests that EV‐DNA–based CNV profiling could complement existing biomarkers such as *BRCA* mutation and HRD status. Further validation in larger, prospective cohorts will be essential to confirm the robustness and clinical applicability of this approach.

In conclusion, this study underscored the importance of EV‐DNA as a promising biomarker for HGSOC in terms of CNV profile. EV‐DNA may provide a stable representation of tumor‐associated genomic information, making it a promising tool for diagnostic and predictive purposes. The five‐gene CNV signature identified in this study showed significant potential as a predictive biomarker for PARP inhibitor response, potentially providing an alternative to existing biomarkers. Further research with larger patient cohorts and improved EV‐DNA enrichment methods would be essential to widely apply CNV analysis in EV‐DNA to clinical use.

## Author Contributions

**Ryosuke Uekusa**: conceptualization, data curation, investigation, writing – original draft, writing – review and editing, visualization, validation, methodology, formal analysis. **Akira Yokoi**: conceptualization, methodology, investigation, project administration, resources, funding acquisition, writing – review and editing, writing – original draft, validation, visualization. **Mayu Ukai**: investigation, resources. **Kosuke Yoshida**: investigation, resources. **Kunanon Chattrairat**: investigation, resources. **Takao Yasui**: investigation, resources. **Yasuhide Inokuma**: supervision. **Masami Kitagawa**: investigation, validation, visualization. **Eri Asano–inami**: investigation, validation. **Masato Yoshihara**: investigation, resources. **Satoshi Tamauchi**: investigation, resources. **Nobuhisa Yoshikawa**: investigation, resources. **Kaoru Niimi**: investigation, resources. **Juntaro Matsuzaki**: investigation, methodology, formal analysis, software, conceptualization, visualization. **Takahiro Ochiya**: supervision. **Yusuke Yamamoto**: investigation, conceptualization. **Hiroaki Kajiyama**: supervision.

## Conflicts of Interest

The authors declare no conflict of interest.

## Supporting information




**Supporting Information**: jev270308‐sup‐0001‐SuppMat.docx

## Data Availability

The data that support the findings of this study are available from the corresponding author upon reasonable request.
