# Supplementary material for: DNA Copy Number Profiling in Extracellular Vesicles as Clinical Biomarkers of High‐Grade Serous Ovarian Carcinoma
Source: J Extracell Vesicles. 2026 May 15;15(5):e70308. doi: 10.1002/jev2.70308 (PMC13178793; doi:10.1002/jev2.70308)
Supplement: Supplementary file 1 — Supporting Information: jev270308‐sup‐0001‐SuppMat.docx [file JEV2-15-e70308-s001.docx]

**Inventory of Supplementary Information**

**Supplementary Figures:**

Supplementary Figure 1-11

**Supplementary Tables:**

Supplementary Table 1-2

**Supplementary figures**

**
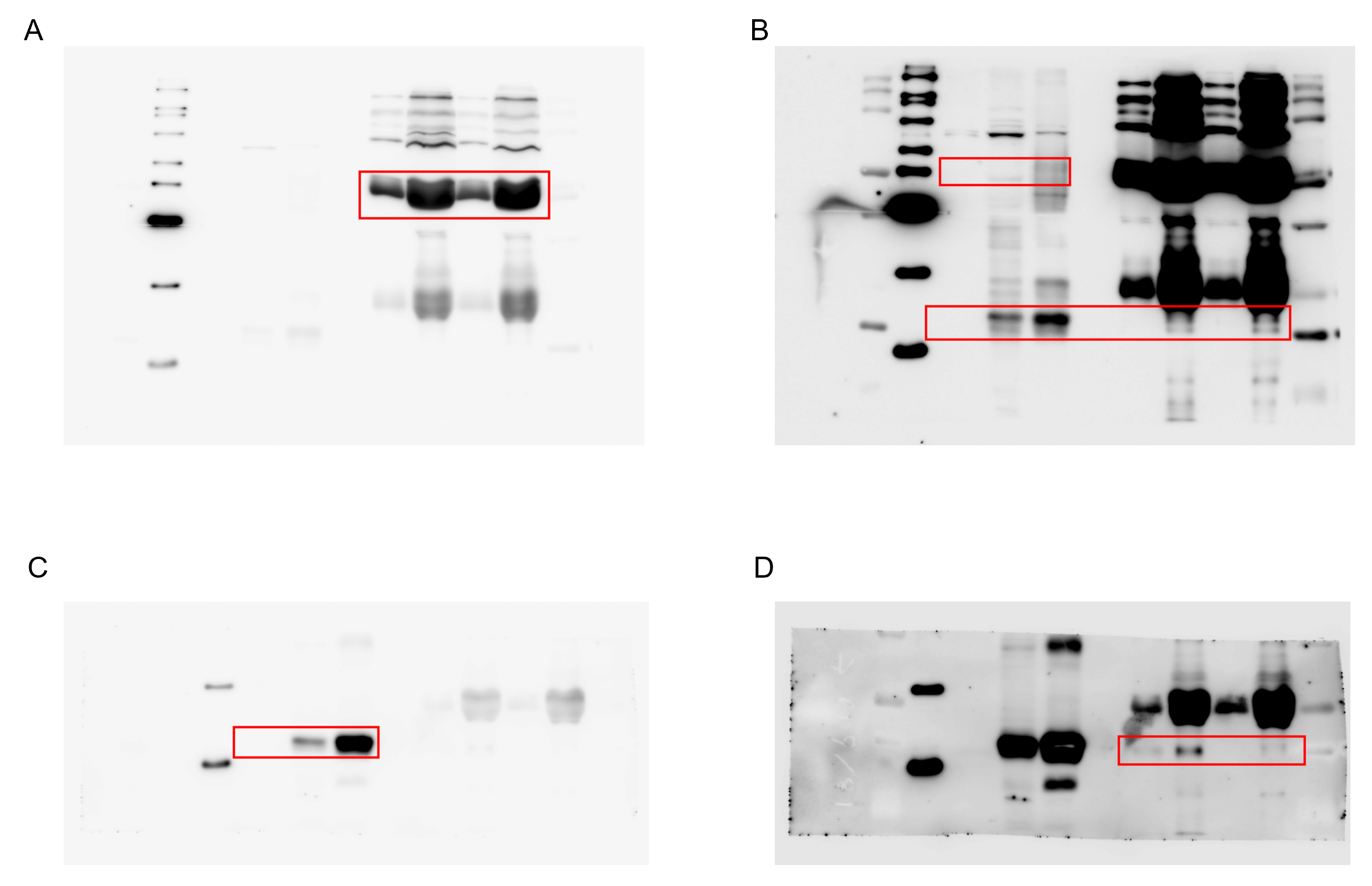
**

**Supplementary Figure 1.** Uncropped Western blot images corresponding to Fig. 1b and Fig. 1f. All lanes and molecular weight markers are shown.

(A) CD9/CD63, short exposure.
(B) CD9/CD63, long exposure.
(C) CD81, short exposure.
(D) CD81, long exposure.

Lane assignments are identical to those shown in the main figures. The regions highlighted by red boxes indicate the areas used in the cropped images presented in Fig. 1b and Fig. 1f. Images with different exposure times are provided to visualize both strong and weak signals. No adjustments were made except for uniform brightness and contrast applied to the entire image.

**Supplementary Figure 2.** The correlations of CNV status between cellular DNA and EV-DNA for KURAMOCHI and OVCAR3. Correlations were assessed by linear regression, and R^2^ indicates the coefficient of determination.

**Supplementary Figure 3.** The correlations of CNV status between tumor DNA and EV-DNA in ascites depending on the starting amount of ascites. Correlations were assessed by linear regression, and R^2^ indicates the coefficient of determination.

**Supplementary Figure 4.** The correlations of CNV status between tumor DNA and EV-DNA in serum from 2 patients with HGSOC. Correlations were assessed by linear regression, and R^2^ indicates the coefficient of determination.

**Supplementary Figure 5.** CNV status of normal ovaries from FFPE tissue (n = 15). DNA was extracted from FFPE tissue of contralateral normal ovarian tissue from patients who had undergone bilateral salpingo-oophorectomy due to benign ovarian tumors.

**Supplementary Figure 6.** The copy numbers of *RAD51*, *BRCA1*, *AKT2*, *CCNE1*, and *MSH6* for normal ovaries (n = 15) and malignant ovarian tumors (n = 14). DNA was extracted from FFPE tissue of normal ovaries from patients who had undergone bilateral salpingo-oophorectomy due to benign ovarian tumors, and from FFPE tissue of malignant ovarian tumors from patients with HGSOC. Comparisons between two groups were performed using the Mann–Whitney U test.

*****p* < 0.0001, ***p* < 0.005, **p* < 0.05

**Supplementary Figure 7.** Comparison of EV–derived DNA levels in ascitic fluid according to cytological classification. Ascitic EV-DNA concentrations were compared among benign, negative, suspicious for malignancy, and positive cytology groups. Data are shown as box-and-violin plots. Data were compared using the Kruskal–Wallis test followed by Dunn’s multiple comparison test.

** indicates *p* < 0.01; *** indicates *p* < 0.001; ns indicates not significant.

**Supplementary Figure 8.** (A) The correlations of CNV status between tumor DNA and total cfDNA. (B) The correlations of CNV status between tumor DNA and total EV-DNA. (C)The correlations of CNV status between tumor DNA and pure cfDNA. Correlations were assessed by linear regression, and R^2^ indicates the coefficient of determination.


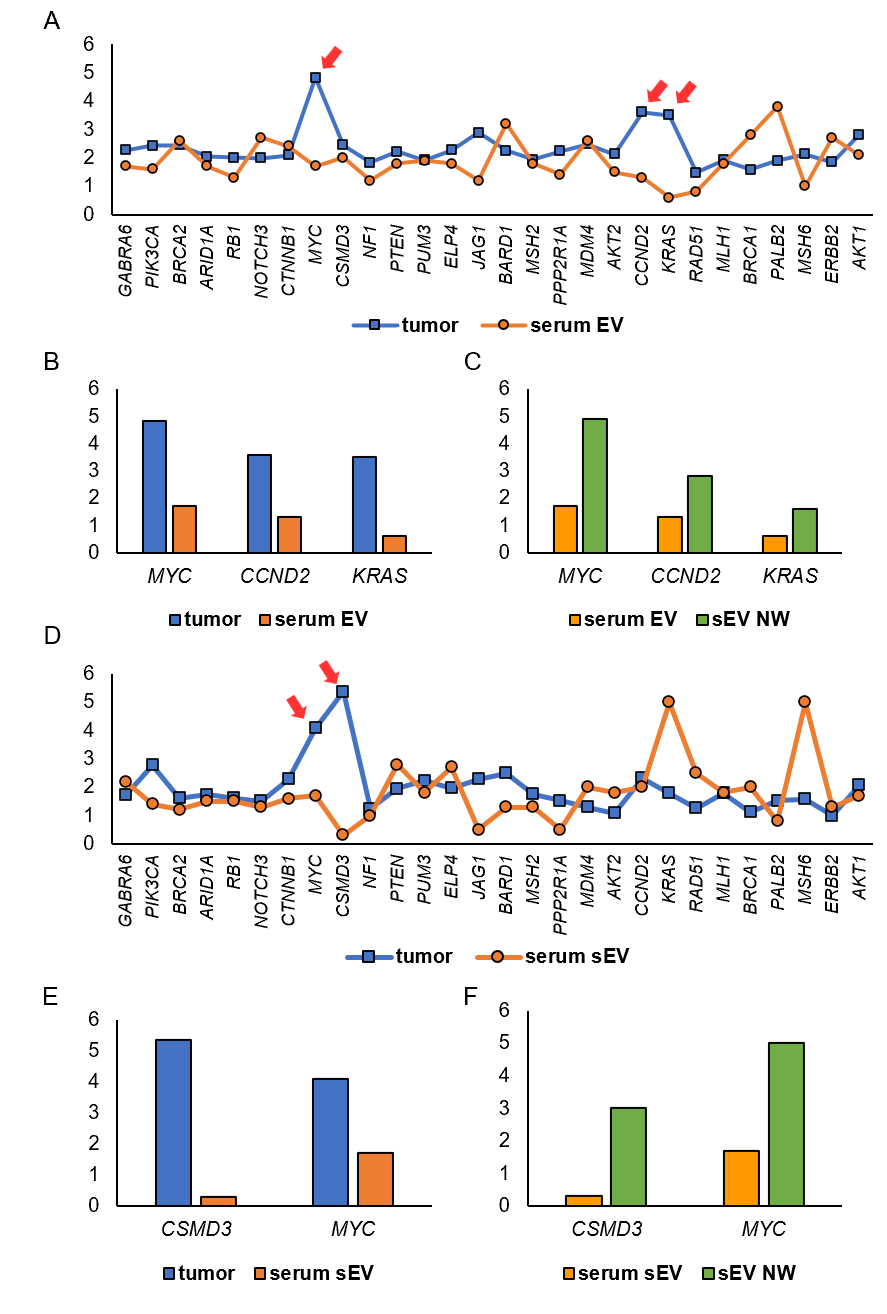


**Supplementary Figure 9.** CNV status of tumor DNA and serum sEV-DNA in two representative cases. (A–C) CNV profiles of tumor DNA and matched serum sEV-DNA in one case. The arrows indicate the top three most amplified genes in tumor DNA. (B, C) Copy numbers of *MYC*, *CCND2*, and *KRAS* in tumor DNA and serum sEV-DNA (B), and in serum sEV-DNA before and after passing through the nanowires (NW) (C). (D–F) CNV profiles of tumor DNA and matched serum sEV-DNA in another case. The arrows indicate the top three most amplified genes in tumor DNA. (E, F) Copy numbers of CSMD3 and MYC in tumor DNA and serum sEV-DNA (E), and in serum sEV-DNA before and after passing through the nanowires (F).


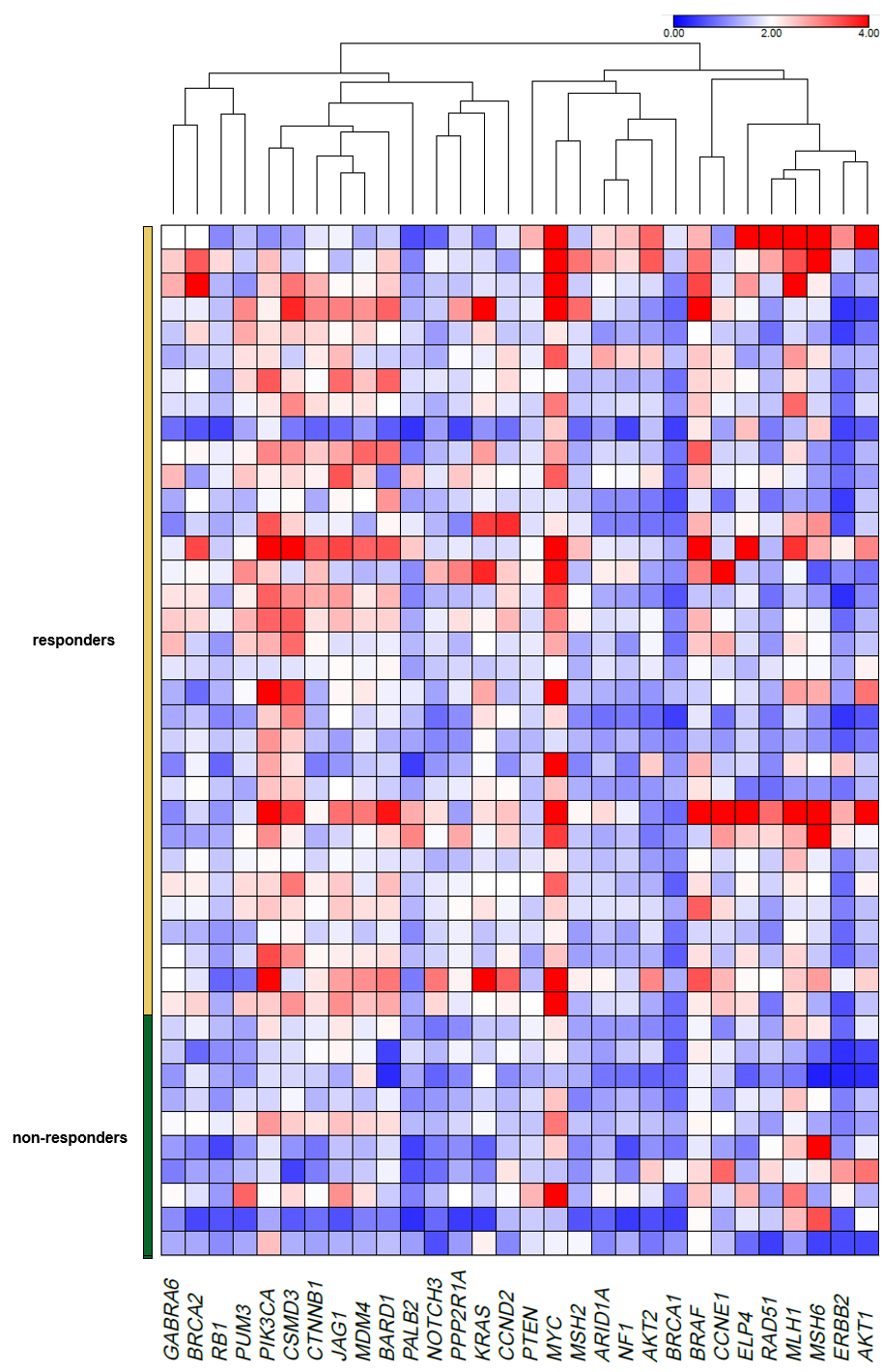


**Supplementary Figure 10.** Hierarchical clustering and heatmap showing the CNV profiles of responders and nonresponders. The copy numbers represent the values before standardization.


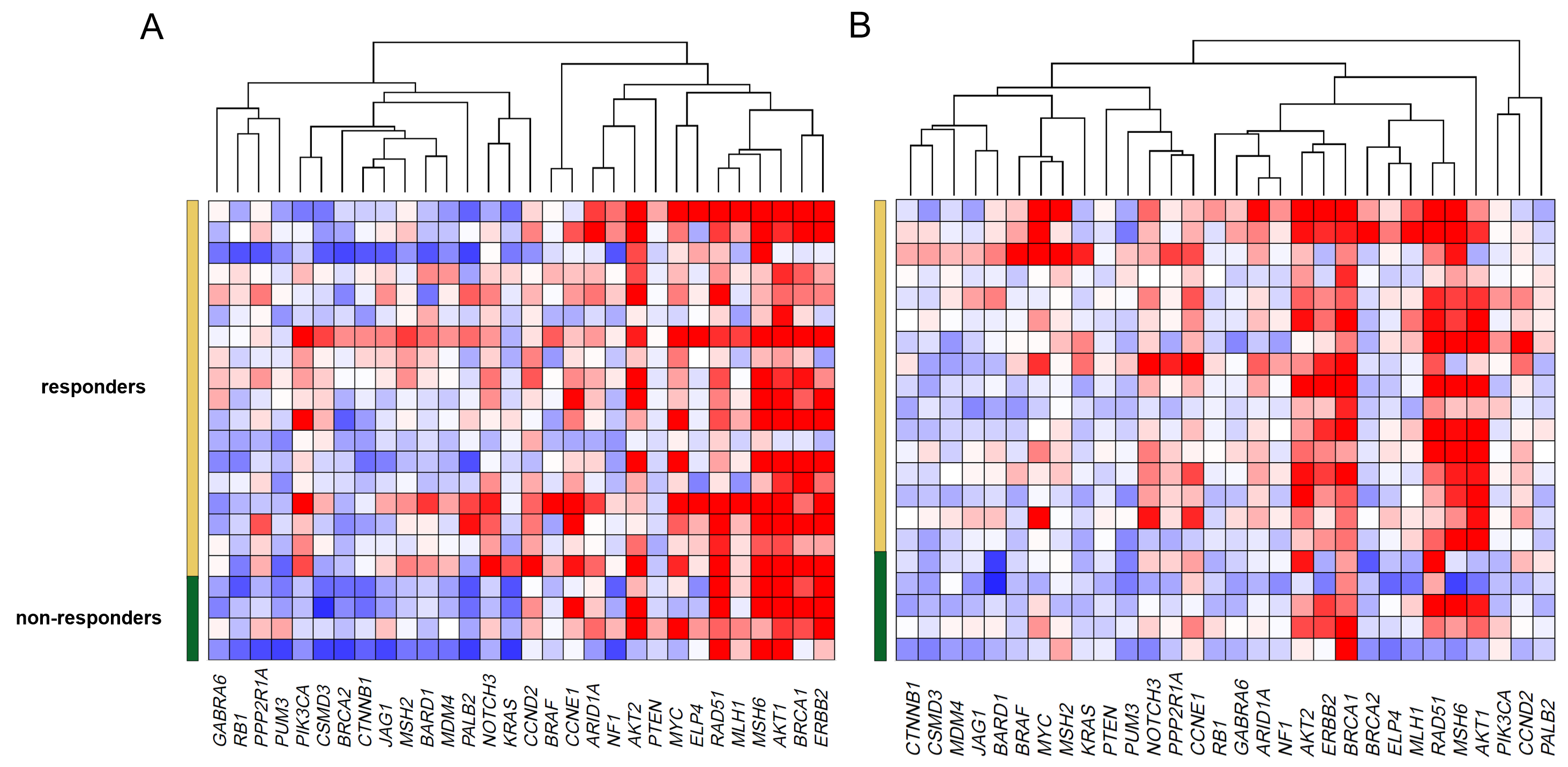


**Supplementary Figure 11.** Hierarchical clustering heatmaps of CNV profiles in responders and nonresponders.
a. CNV profiles from tumor DNA of chemotherapy-naïve patients.
b. CNV profiles from tumor DNA of patients with prior chemotherapy.

**Supplementary Tables**

**Supplementary table 1. Patient characteristics for CNV analysis using FFPE samples of contralateral normal ovarian tissue obtained at surgery for ovarian tumor**

| Pt. number | Age | Histological type |
| --- | --- | --- |
| N1 | 69 | Serous cystadenoma |
| N2 | 63 | Serous cystadenoma |
| N3 | 45 | Serous cystadenoma |
| N4 | 75 | Benign cyst |
| N5 | 48 | Mucinous cystadenoma |
| N6 | 52 | Mucinous adenofibroma |
| N7 | 70 | Benign cyst |
| N8 | 48 | Endometriotic cyst |
| N9 | 72 | Serous cystadenoma |
| N10 | 65 | Serous adenofibroma |
| N11 | 60 | No tumor |
| N12 | 88 | Serous cystadenoma |
| N13 | 56 | Benign cyst |
| N14 | 73 | Fibroma |
| N15 | 77 | Serous cystadenoma |
| C1 | 56 | HGSOC |
| C2 | 68 | HGSOC |
| C3 | 63 | HGSOC |
| C4 | 49 | HGSOC |
| C5 | 59 | HGSOC |
| C6 | 72 | HGSOC |
| C7 | 33 | HGSOC |
| C8 | 53 | HGSOC |
| C9 | 60 | HGSOC |
| C10 | 63 | HGSOC |
| C11 | 70 | HGSOC |
| C12 | 38 | HGSOC |
| C13 | 77 | HGSOC |
| C14 | 60 | HGSOC |
| N; normal, C; Cancer, HGSOC; High-grade serous ovarian carcinoma | | |

**Supplementary table 2. Patient characteristics for EV-DNA analysis by ascites cytology**

| **Category** | **Subcategory** | **B group (N=26)** | **N group (N=32)** | | | **P group (N=36)** | |
| --- | --- | --- | --- | --- | --- | --- | --- |
| Histological type | Fibroma / fibrothecoma | 12 | - | | | - | |
|  | Endometrial cyst | 6 | - | | | - | |
|  | Mucinous cystadenoma | 5 | - | | | - | |
|  | Others | 3 | - | | | - | |
|  | High-grade serous carcinoma | - | 3 | | | 25 | |
|  | Endometrial carcinoma | - | 13 | | | 4 | |
|  | Clear cell carcinoma | - | 11 | | | 5 | |
|  | Mucinous carcinoma | - | 5 | | | 2 | |
| Cytology | Negative | 26 | 22 | | | 0 | |
|  | Suspicious | 0 | 10 | | | 0 | |
|  | Positive | 0 | 0 | | | 36 | |
| FIGO stage (2014) | Stage I | - | 23 | | | 2 | |
|  | Stage II | - | 2 | | | 1 | |
|  | Stage III | - | 5 | | | 16 | |
|  | Stage IV | - | 2 | | | 17 | |
| Age (years) |  | 57 (28–87) | 49 (25–87) | | | 58 (37–85) | |
| B; Benign, N; Cytology Negative (malignant), P; Cytology Positive (malignant) | | | |  |  | |  |
